# Supplementary material for: Low-Mineral Water Diminishes the Bone Benefits of Boron
Source: Nutrients. 2024 Aug 28;16(17):2881. doi: 10.3390/nu16172881 (PMC11397211; doi:10.3390/nu16172881)
Supplement: Supplementary file 1 [file nutrients-16-02881-s001.zip › nutrients-3126666-supplementary.pdf]

Supplemental Material: Low-Mineral Water Diminishes the Bone Benefits of Boron

Ting Huang [1,†], Yuhui Hao [2,†], Yao Tan [1], Qijie Dai [3], Weiyan Chen [1], Ke Cui [1], Jiaohua Luo [1], Hui Zeng [1], Weiqun Shu [1,*] and Yujing Huang [1,*]

1 Department of Environmental Hygiene, College of Preventive Medicine, Army Medical University, Chongqing 400038, China; huangting08123@163.com (T.H.); xiaoyue7122@tmmu.edu.cn (Y.T.); weiyanchen@tmmu.edu.cn (W.C.); cuike@tmmu.edu.cn (K.C.); ljh978@tmmu.edu.cn (J.L.); zenghui@tmmu.edu.cn (H.Z.)
2 State Key Laboratory of Trauma and Chemical Poisoning, Institute of Combined Injury, Chongqing Engineering Research Center for Nanomedicine, College of Preventive Medicine, Army Medical University, Chongqing 400038, China; yuhuihao@tmmu.edu.cn
3 Department of Orthopedics, Southwest Hospital, Army Medical University, Chongqing 400038, China; mapaler@163.com
* Correspondence: weiqunshu@tmmu.edu.cn (W.S.); huangyujing@tmmu.edu.cn (Y.H.)
† These authors contributed equally to this work.

Supplementary Figures

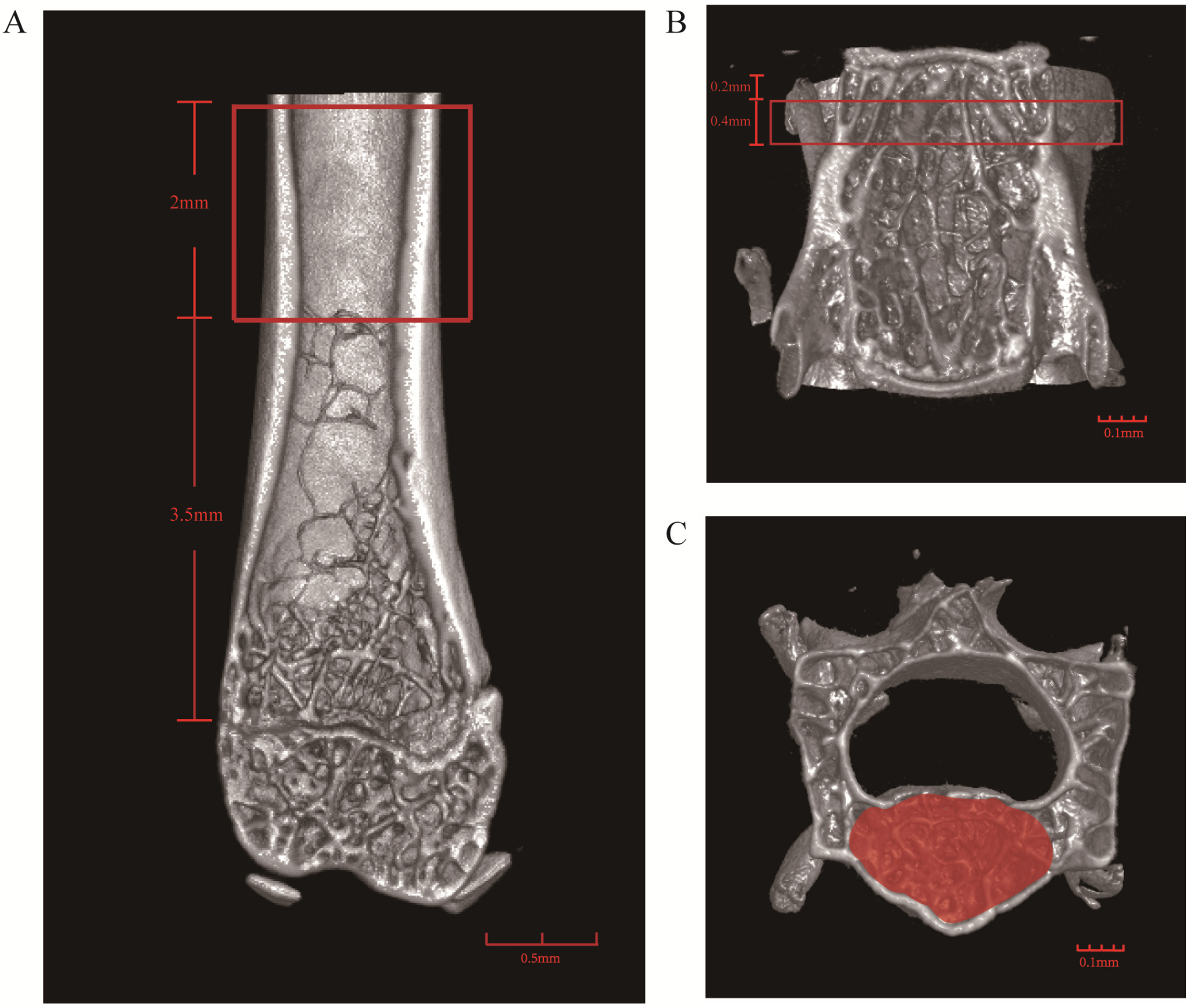

Figure S1. Regions of interest (ROIs) of cortical bone for the femur (**A**) and trabecular bone for the lumbar vertebra (**B**,**C**). (**A**) images of the femur in the horizontal plane. (**B**) images of the lumbar vertebra in the coronal plane. (**C**) images of the lumbar vertebra in the horizontal plane.

Supplementary Tables

Table S1. Daily mineral intake in mice in 14th week.

	Dietary Intake [1]			Intake from Drinking Water [1]			Total Intake [1]		
	**Tap Water [2]**	**Purified Water [3]**	***p***	**Tap Water [2]**	**Purified Water [3]**	***p***	**Tap Water [2]**	**Purified Water [3]**	***p***
	$n = 4$ [4]	$n = 4$ [4]		$n = 4$ [4]	$n = 4$ [4]		$n = 4$ [4]	$n = 4$ [4]	
Ca, μg/d	26563.00 ±282.68	25079.56 ±577.76	0.077	124.04 ±4.96	0.00 ±0.00	<0.001	26687.04±279.41	25079.56±577.76	0.062
Mg, μg/d	7627.00 ±81.17	7201.06 ±165.89	0.077	23.47 ±0.94	0.00 ±0.00	<0.001	7650.47±80.55	7201.06±165.89	0.066
P, μg/d	17884.00 ±190.32	16885.25 ±388.99	0.077	0.34 ±0.01	0.00 ±0.00	<0.001	17884.34±190.31	16885.25±388.99	0.077
Ratio of Ca: Mg	3.48	3.48		5.29	—		3.49	3.48	

[1] Values are means ± SEM.

[2] All mice drank tap water with or without boron exposure.

[3] All mice drank purified water with or without boron exposure.

[4] Four groups of mice, each with only one set of food or water intake data.

Table S2. Daily boron intake in mice in 14th week.

Type of Water Comsuming	Boron Exposure (mg/L)	Dietary Intake (μg/d)	Intake from Drinking Water (μg/d)	Total Intake (μg/d)	The Drinking Water's Contribution to Total Intake (%)
Tap water [1]	0	81.62	0.06	81.68	0.07
	5	82.54	16.01	98.55	16.25
	40	81.31	143.27	224.58	63.79
	200	78.54	716.07	794.61	90.12
Purified water [2]	0	73.3	0	73.3	0
	5	80.08	17.38	97.46	17.83
	40	73.61	122	195.61	62.37
	200	78.93	713.75	792.68	90.04
Mean [3]	0	77.46	0.03	77.49	0.035
	5	81.31	16.695	98.005	17.04 [a]
	40	77.46	132.635 [ab]	210.095 [ab]	63.08 [ab]
	200	78.735	714.91 [abc]	793.645 [abc]	90.08 [abc]

[1] All mice drank tap water with or without boron exposure.
[2] All mice drank purified water with or without boron exposure.
[3] Mean of two groups exposed to the same dose of boron.
[a] There is a significant difference compared with the groups without boron exposure ($p < 0.05$).
[b] There is a significant difference compared with the groups exposed to 5 mg/L boron ($p < 0.05$).
[c] There is a significant difference compared with the groups exposed to 40 mg/L boron ($p < 0.05$).
